# Chemomodulation of human dendritic cell function by antineoplastic agents in low noncytotoxic concentrations

**DOI:** 10.1186/1479-5876-7-58

**Published:** 2009-07-10

**Authors:** Ramon Kaneno, Galina V Shurin, Irina L Tourkova, Michael R Shurin

**Affiliations:** 1 Department of Microbiology and Immunology, Institute of Biosciences, São Paulo State University, Botucatu, SP, Brazil; 2 Departments of Pathology, University of Pittsburgh Medical Center, Pittsburgh, PA, USA; 3 Department of Immunology, University of Pittsburgh Medical Center, Pittsburgh, PA, USA

## Abstract

The dose-delivery schedule of conventional chemotherapy, which determines its efficacy and toxicity, is based on the maximum tolerated dose. This strategy has lead to cure and disease control in a significant number of patients but is associated with significant short-term and long-term toxicity. Recent data demonstrate that moderately low-dose chemotherapy may be efficiently combined with immunotherapy, particularly with dendritic cell (DC) vaccines, to improve the overall therapeutic efficacy. However, the direct effects of low and ultra-low concentrations on DCs are still unknown. Here we characterized the effects of low noncytotoxic concentrations of different classes of chemotherapeutic agents on human DCs *in vitro*. DCs treated with antimicrotubule agents vincristine, vinblastine, and paclitaxel or with antimetabolites 5-aza-2-deoxycytidine and methotrexate, showed increased expression of CD83 and CD40 molecules. Expression of CD80 on DCs was also stimulated by vinblastine, paclitaxel, azacytidine, methotrexate, and mitomycin C used in low nontoxic concentrations. Furthermore, 5-aza-2-deoxycytidine, methotrexate, and mitomycin C increased the ability of human DCs to stimulate proliferation of allogeneic T lymphocytes. Thus, our data demonstrate for the first time that in low noncytotoxic concentrations chemotherapeutic agents do not induce apoptosis of DCs, but directly enhance DC maturation and function. This suggests that modulation of human DCs by noncytotoxic concentrations of antineoplastic drugs, i.e. chemomodulation, might represent a novel approach for up-regulation of functional activity of resident DCs in the tumor microenvironment or improving the efficacy of DCs prepared *ex vivo *for subsequent vaccinations.

## Introduction

Chemotherapy is the treatment of choice for most patients with inoperable and advanced cancers and more than half of all people diagnosed with cancer receive chemotherapy. Chemotherapy is also often used as neoadjuvant or adjuvant modality for preoperative or postoperative treatment, respectively [1]. The antineoplastic chemotherapeutic agents belong to several groups according to the mechanism of their action, which include antimicrotubule and alkylating agents, anthracyclines, antimetabolites, topoisomerase inhibitors, plant alkaloids, and others [2].

Based on pre-clinical experiments, the log-dose survival curve model for cancer cell killing became the leading model for chemotherapy dose calculation [3]. The dose-delivery schedule of conventional chemotherapy, which determines its efficacy and toxicity, is based on the maximum tolerated dose (MTD), i.e. the highest dose of a drug that does not cause unacceptable side effects. This strategy of MTD chemotherapy has lead to cure and disease control in a significant number of patients but is associated with significant short-term and long-term toxicity and complications, including myelosuppression, neutropenia, trombocytopenia, increased risk of infection and bleeding, gastrointestinal dysfunctions, arthralgia, liver toxicity, and the cardiac and nervous system damage [4-6].

Recent studies have shown that cytotoxic drugs used at lower doses (10–33% of the MTD) and given more frequently – low-dose metronomic chemotherapy or a 'lower' dose dense chemotherapy, may have the potential for antitumor efficacy by inhibiting tumor angiogenesis [7,8]. Although low-dose metronomic chemotherapy can lead to a significant response rate and stable disease in certain patient populations, this approach can be associated with chronic toxicity such as severe lymphopenia with opportunistic infection [3]. Interestingly, moderately low-dose chemotherapeutics, for instance anthracyclins, have been recently reported to indirectly activate dendritic cells (DCs) by inducing secretion of alarmin protein from dying tumor cells [9,10]. DCs, the most powerful antigen-presenting cells, play a key role in induction and maintenance of antitumor immunity and are widely tested as promising therapeutic cancer vaccines in multiple ongoing clinical trials [11]. However, other studies demonstrated that many chemotherapeutic drugs in conventional or moderately low concentrations could induce apoptosis of DCs, directly inhibit their maturation and function, expression of co-stimulatory molecules, suppress dendropoiesis, and polarize DC development *in vitro *as well as *in vivo *in chemotherapy-treated patients [12-19]. We have recently reported that several chemotherapeutic agents could directly modulate key signaling pathways [20] and production of IL-12, IL-10, IL-4, and TNF-α [21] in murine DCs without inducing apoptotic death of DCs when used in ultra-low noncytotoxic concentrations. Further investigation of this phenomenon, which can be termed *chemomodulation*, revealed that certain chemotherapeutic agents from different groups in low noncytotoxic concentrations directly up-regulated maturation, expression of co-stimulatory molecules, and processing and presentation of antigens to antigen-specific T cells by murine DCs [22]. Although indirect activation of human DCs by signals expressed on or released by dying tumor cells due to chemotherapy, such as calreticulin, heat-shock proteins, HMGB1, alarmin, and uric acid, can be predicted [23-25], it is still unclear whether chemotherapeutic agents in noncytotoxic concentrations might directly modulate the activity of human DCs.

Recent data demonstrate that administration of chemotherapeutic agents in conventional or low doses might significantly attenuate the antitumor potential of DC vaccines. For instance, gemcitabine increased survival of mice treated with DC-based vaccines in a pancreatic carcinoma model [26]. In murine fibrosarcoma model, combined treatment of paclitaxel chemotherapy and the injection of DCs led to complete tumor regression, in contrast to only partial eradication of the tumors with chemotherapy or DCs alone [27]. We have recently reported that low-dose paclitaxel markedly up-regulates antitumor immune responses in mice bearing lung cancer and treated with DC vaccines [28]. Given the fact that DC vaccines combined with chemotherapy show therapeutic feasibility [29] and are highly applicable for human treatment [30], the goal of these studies was to determine whether FDA-approved chemotherapeutic agents in low noncytotoxic concentrations might directly affect viability, maturation, and function of human DCs *in vitro*. Our data demonstrate that certain chemotherapeutic agents in low noncytotoxic concentrations do not alter viability of human tumor cell lines or human DCs, but directly augment phenotypic maturation and antigen-presenting potential of DCs. This suggests that chemomodulation, i.e. modulation of DC function by noncytotoxic concentrations of antineoplastic drugs, might represent a novel approach for improving the functional activity of DCs in the tumor microenvironment and increasing the efficacy of DC-based vaccination protocols.

## Materials and methods

### Antineoplastic chemotherapeutic agents

The following chemotherapeutic agents were used (with the commercial brand names): the antimicrotubule agents vinblastine (Velban), vincristine (Oncovin), and paclitaxel (Taxol); the antimetabolites 5-aza-2-deoxycytidine (Vidaza) and methotrexate (Rheumatrex, Trexall); the alkylating agents cyclophosphamide (Cytoxan) and mitomycin C (Mutamycin); the topoisomerase inhibitor doxorubicin (Adriamycin); the platinum agents cisplatin (Platinol) and carboplatin (Paraplatin); the hormonal agents flutamide (Drogenil, Eulexin) and tamoxifen (Nolvadex); and the cytotoxic glycopeptide antibiotic bleomycin (Blenoxane). 5-Bleomycin and 5-aza-deoxycytidin were purchased from Sigma-Aldrich (St. Louis, USA) and paclitaxel – from F.H. Faulding & Co. Ltd. (Mulgrave, Autralia). All other drugs were purchased form Calbiochem (La Jolla, USA). All drugs were first dissolved in endotoxin-free water following by appropriated dilutions in culture medium as stated.

### Establishing noncytotoxic concentrations of chemotherapeutic drugs

Dose-dependent cytotoxicity of tested drugs was initially tested on the following human tumor cell lines: LNCaP prostate adenocarcinoma (ATCC, Manassas, VA, USA), PCI-4B head and neck squamous cell carcinoma (UPCI, Pittsburgh, PA, USA), and HCT-116 and HT-29 colon adenocarcinomas (ATCC). Cells were cultured in RPMI 1640 medium supplemented with 10% FBS, 2 mM L-glutamine, 1 mM sodium pyruvate, 0.1 mM nonessential amino acids, 10 mM HEPES, and 0.1 mg/ml gentamicin (complete medium, CM) at 37°C and 5% CO_2_. All cell lines were *Mycoplasma*-free.

The Effective Concentration (EC) of each of the tested chemotherapeutic agent, i.e. the highest concentration of a chemotherapeutic agent that does not inhibit the proliferative activity of tumor cells, was determined by the modified MTT cytotoxicity assay. Briefly, tumor cells (2 × 10^4 ^cells/ml) were cultured in 96-well flat-bottom plates (100 μl/well) for 24 h. After attachment, cells were treated with different concentrations of tested drugs (0–100,000 nM) for 48 h. Then, the plates were centrifuged and 100 μl of supernatant in each well were replaced with 100 μl of (3-(4,5-Dimethylthiazol-2-yl)-2,5-diphenyltetrazolium bromide bromide (MTT, Sigma) solution (1 mg/ml). Cells were cultured for 3 h, the supernatants were removed and 100 ul of dimethylsulphoxide (DMSO) was added to each well to dissolve MTT. Plates were read at 540 nm (Wallac Microplate reader, Turku, Finland) and EC values were estimated based on the MTT reduction to formazan in living cells. Cells were considered resistant to the treatment if corresponding EC values were greater than 1,000 nM.

### Generation of human monocyte-derived DCs

Human DCs were prepared from peripheral blood mononuclear cells (PBMCs) of healthy donors as described earlier [31]. Briefly, after gradient separation on Lymphoprep-1077 (Axes Shield PoC, Oslo, Norway) and lysis of red blood cells, PBMCs were resuspended in AIM-V medium (Invitrogen Co., Carlsbad, USA) and seeded in 6-well plates (10^7^cells/well). After incubation for 60 min at 37°C, non-adherent cells were removed, and adherent monocytes were cultured in CM with 1000 U/ml recombinant human (rh) GM-CSF and 1000 U/ml rhIL-4 (PeproTech, Rocky Hill, USA). Chemotherapeutic agents were added to DC cultures on day 1, DCs were harvested on day 6 and DC phenotype and function, as well as signs of apoptosis were characterized as described below.

### Evaluation of DC apoptosis induced by chemotherapeutics

Drug-induced apoptosis of DCs was assessed by the Annexin V binding assay, as described earlier [20]. Cells were stained with FITC-Annexin V (BD-PharMingen, San Diego, USA) and propidium iodide (PI, 10 μg/ml, Sigma). Cells undergoing early apoptosis were determined as the percentage of Annexin V^+^/PI^- ^cells by FACScan with Cell Quest 1.0 software package (BD, San Diego, USA). Detection of early apoptotic events in DCs was shown to be a more sensitive approach to estimate noncytotoxic concentrations of chemotherapeutic agents than evaluation of both apoptotic/necrotic events as Annexin V+/PI+ cells. Thus, the results are shown as the mean percentage of Annexin V+/PI- cells ± SEM.

### Analysis of DC phenotype

Control non-treated and drug-treated DCs were washed in PBS containing 0.1% BSA and analyzed by flow cytometry as described earlier [32]. Monoclonal antibodies (BD-Pharmingen) against human HLA-DR, HLA-ABC, CD83, CD80, CD86, CD40, and CD1a conjugated with FITC or PE were added to cells and incubated for 30 minute at 4°C. Murine FITC-IgG and PE-IgG were used as isotype controls. Data analysis was performed using the CellQuest and WinMDI software and the results were expressed as the percentage of positive cells or Mean Fluorescent Intensity (MFI).

### Mixed leukocyte reaction (MLR)

Functional activity of DCs was assessed by measuring their ability to stimulate proliferation of allogeneic T lymphocytes isolated from PBMCs of healthy volunteers [33]. Drug-treated and control DCs were co-cultured with allogeneic nylon wool-enriched T lymphocytes in a 96-round bottom plates at different DC:T ratios (1:1, 1:3, 1:10, 1:30, 1:100, and 1:300) in 200 μl of CM for 96 h. Cultures were pulsed with ^3^H-thymidine (1 μCi/well, Perkin Elmer, Boston, USA) for 4 h and harvested onto glass fiber filters GF/C (Wallac, Turku, Finland). Uptake of ^3^H-thymidine was assessed on liquid scintillation counter (Wallac 1205 Betaplate) and the results were expressed as count per minute (cpm).

### Statistical analysis

The effect of tested drugs on tumor cells and DCs viability was analyzed by Student's *t *test comparing each group with untreated controls. Alterations in DC phenotype and MLR activity were evaluated by Kruskal-Wallis one-way ANOVA. The differences were considered significant when error probability was less than 5% (p < 0.05). All statistical analyses were done using SigmaPlot 11.0 software (SSNS).

## Results

### Noncytotoxic concentration of chemotherapeutic agents

Determination of noncytotoxic concentrations of 13 antineoplastic drugs was done using four human tumor cell lines by examining viability of cells treated with a drug in different concentrations (0 – 100 μM). The highest concentrations that do not inhibit tumor cell proliferation are shown in Table 1 as the results of three independent experiments. As can be seen, the effective concentrations of tested agents differ for different tumor cell lines. For instance, prostate cancer cells showed the highest resistance to tested cytotoxic agents, while colon cancer cell lines were relatively sensitive. Interestingly, both LNCaP and PCI-4B cell were resistant to the effects of platinum and hormonal agents. These results thus allowed exclusion of five chemotherapeutic agents (cyclophosphamide, cisplatin, carboplatin, flutamide, and tamoxifen) from further analysis since these agents did not display a dose-dependent cytotoxicity against selected tumor cell lines. The ability of the remaining chemotherapeutic agents to induce dose-dependent cytotoxic effect on human DCs was evaluated in the next series of experiments.

**Table 1:** Noncytotoxic concentrations of chemotherapeutic agents (MTT assay)

Chemotherapeutic agents	EC* LNCaP	EC PCI-4B	EC HCT-116	EC HT-29
***Antimicrotubule agents***				
Vinblastine (Velban)	100 nM	10 nM	ND	ND
Vincristine (Oncovin)	100 nM	0.1 nM	ND	ND
Paclitaxel (Taxol)	10 nM	0.1 nM	0.5 nM	5 nM

***Antimetabolites***				
5-azacytidine (Vidaza)	100 nM	50 nM	ND	ND
Methotrexate (Rheumatrex, Trexall)	5 nM	5 nM	0.5 nM	ND

***Alkylating agents***				
Cyclophosphamide (Cytoxan)	Resistant**	50 nM	50 nM	ND
Mitomycin C (Mutamycin)	500 nM	50 nM	ND	ND

***Topoisomerase inhibitors***				
Doxorubicin (Adriamycin)	100 nM	50 nM	5 nM	5 nM

***Platinum agents***				
Cisplatin (Platinol)	Resistant	Resistant	ND	ND
Carboplatin (Paraplatin)	Resistant	Resistant	ND	ND

***Hormonal agents***				
Flutamide (Drogenil, Eulexin)	Resistant	Resistant	ND	ND
Tamoxifen (Nolvadex)	1000 nM	Resistant	ND	ND

***Others***				
Bleomycin (Blenoxane)	100 nM	100 nM	ND	ND

*, EC, Effective concentration – the maximal concentration of a chemotherapeutic agent that caused no inhibition of tumor cell activity in the MTT assay.

**, Cells were considered resistant to the treatment when the EC value was greater than 1,000 nM.

LNCaP, human prostate cancer cell line; PCI-4B, human head and neck squamous cell carcinoma cell line; HCT-116 and HT-29, human colon cancer cell lines; MTT, (3-(4,5-Dimmethylthiazol-2-yl)-2,5-diphenyltetrazolium bromide) reduction assay; ND, not determined.

DC response to the cytotoxic effect of chemotherapeutics cannot be determined in the MTT assay because many drugs in low and moderately low concentrations induce activation of mitochondrial dehydrogenases in DCs, which makes the analysis of dose-dependent cell viability unfeasible. Therefore, we utilized Annexin V/PI staining to establish noncytotoxic concentrations of eight chemotherapeutic agents for human DCs. Cells were treated with a range of concentrations of cytotoxic agents (0–100 nM) and the levels of apoptosis were assessed by Annexin V/PI binding assay (Table 2). The results showed that the EC values of tested drugs for the tumor cell lines were similar to or lower than the EC values for DCs, suggesting that tumor cells are more sensitive to tested substances than DCs are. These data allowed the establishment of concentrations of chemotherapeutic drugs that are nontoxic for tumor cell lines and DCs. To ensure that no cytotoxicity is induced in experiments determining the effects of drugs on DC phenotype and function *in vitro*, we used the concentrations of drugs that are even 5–10-fold lower than those established in Tables 1 and 2.

**Table 2:** Sensitivity of human DCs to the cytotoxic effects of antineoplastic chemotherapeutic agents *in vitro*

Chemotherapeutic agent (concentration, nM)	Apoptosis of DCs (% ± SEM)
vinblastine (50)	3.2 ± 0.9
vinblastine (10)	1.1 ± 1.3
vinblastine (1)	0.9 ± 0.3
vinblastine(0.1)	-0.6 ± 0.3

vincristine (50)	6.5 ± 2.1
vincristine (10)	3.3 ± 1.9
vincristine (1)	0.5 ± 0.6
vincristine (0.1)	-0.6 ± 0.9

paclitaxel (25)	4.9 ± 2.3
paclitaxel (5)	2.2 ± 0.7
paclitaxel (1)	2.2 ± 0.4
paclitaxel (0.1)	0.1 ± 0.3

5-aza-2deoxycitidine (25)	7.4 ± 3.3
5-aza-2deoxycitidine (5)	0.8 ± 0.8

methotrexate (25)	3.9 ± 1.1
methotrexate (5)	0.8 ± 0.6
methotrexate (1)	0.3 ± 0.4

mitomycin C (25)	1.3 ± 1.4
mitomycin C (5)	-0.9 ± 0.4

doxorubicin (100)	5.5 ± 0.9
doxorubicin (25)	3.4 ± 0.3
doxorubicin (5)	0.6 ± 1.8

Analysis of DC survival was carried out by flow cytometry after the staining with FITC-Annexin V and propidium iodide. DCs were treated with the cytotoxic agents for 48 h and analyzed by FACScan after staining. The background staining of control non-treated DC value was subtracted from experimental results. The results are express as the mean percentage of Annexin+PI- cells ± SEM of 3 independent assays. Student's *t *test was applied to compare the results of the treatment with different drug concentrations with control non-treated DC values in order to determine Effective Concentration (EC), i.e. the highest concentration of a chemotherapeutic agent that does not induce apoptosis in DCs.

### Chemomodulation of DC phenotype by low noncytotoxic concentrations of chemotherapeutic agents

Phenotype of control and drug-treated DCs was analyzed by the expression of HLA-DR, CD83, CD80, CD86, CD40, and CD1a molecules. The results in Table 3 show that vincristine, vinblastine, paclitaxel, mitomycin C, and doxorubicin markedly (25–70%) increased the expression of CD83 molecules on DC surface, suggesting up-regulation of DC maturation. The results in Table 3, calculated from MFI values, are expressed as the percentage of MFI increase in drug-treated DCs in comparison to MFI values in control untreated DCs. Increase in expression of an assessed marker of greater than 30% was considered to be biologically significant and was examined with the statistical analysis. Although the results were donor-dependent, the up-regulation of CD83 expression on DCs treated with vinblastine, paclitaxel, and doxorubicin was statistically significant (p < 0.05). For instance, vinblastine elevated expression of CD83 on DCs in 2.5-fold increasing it from 3.37 MFI to 8.16 MFI in healthy Donor 1. Furthermore, DCs treated with antimicrotubule agents vinblastine, vincristine and paclitaxel, and antimetabolites azacytydine and methotrexate displayed enhanced expression of CD40 molecules (up to 30–50%, p < 0.05). For instance, in Donor 1, methotrexate doubled expression of CD40 rising it from 12.44 MFI to 20.19 MFI, while in donor 3 expression of CD40 was increased from 90.04 MFI to 130.11 MFI. Interestingly, expression of HLA-DR and CD86 molecules on DCs was not markedly altered by tested chemotherapeutic agents in low noncytotoxic concentrations, although in donor 3, vinblastine and azacytidine up-regulated expression of MHC class II molecules up to 50%. Altogether, these results demonstrate that, in spite of the fact that stimulation of expression of MHC class II and co-stimulatory molecules on DCs was drug- and donor-dependent, many of the tested chemotherapeutic drugs were able to directly up-regulate maturation of human DCs *in vitro*.

**Table 3:** Chemomodulation of phenotypic maturation of human DCs *in vitro*

Marker	HLA-DR	CD83	CD80	CD86	CD40	CD1a
Agent						
**Vinblastine, 1 nM**	25.0 ± 4.5	72.7 ± 10.1*	16.5 ± 13.1	2.9 ± 3.5	46.1 ± 3.1*	16.7 ± 0.9

**Vincristine, 1 nM**	10.7 ± 0.7	25.9 ± 4.9	1.6 ± 0.2	27.0 ± 22.0	52.7 ± 2.9*	19.4 ± 0.3

**Paclitaxel, 5 nM**	0.5 ± 3.1	30.2 ± 5.7*	5.4 ± 27.5	6.9 ± 3.2	29.3 ± 3.4*	6.0 ± 2.3

**5-aza-2-deoxycytidine, 5 nM**	29.1 ± 12.2	8.1 ± 4.2	50.2 ± 3.2*	2.4 ± 6.4	33.4 ± 6.9*	10.8 ± 4.3

**Methotrexate, 5 nM**	3.6 ± 2.2	2.1 ± 1.8	6.5 ± 3.6	6.2 ± 0.8	51.0 ± 6.5*	35.9 ± 5.7*

**Mitomycin C, 50 nM**	4.2 ± 2.7	25.0 ± 12.8	12.0 ± 22.2	3.4 ± 0.9	24.9 ± 12.3	32.1 ± 5.8*

**Doxorubicin, 10 nM**	4.7 ± 0.3	38.8 ± 4.3*	4.24 ± 5.9	3.1 ± 2.0	14.3 ± 6.8	5.3 ± 7.1

The results in Table 3, calculated from MFI values, are expressed as the percentage of MFI increase in drug-treated DCs in comparison to MFI in untreated DCs. Increase in any marker expression of greater than 30% was considered to be biologically significant and was analyzed for statistical significance of changes. Data represent the mean ± SEM from 3 independent experiments utilizing cells from 3 different healthy donors. *, p < 0.05 (ANOVA, N = 3).

**Figure 1:**
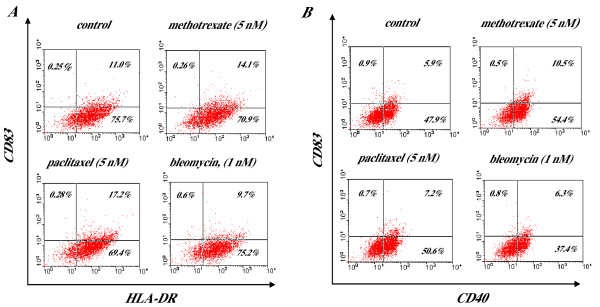
**Chemomodulation of phenotype of human DCs by antineoplastic chemotherapeutic agents in low noncytotoxic concentrations**. DCs were generated from monocyte isolated from PBMC of healthy volunteers by culturing monocytes in complete medium supplemented with GM-CSF and IL-4 as described in Materials and Methods. Chemotherapeutic agents were added to DC cultures for 48 h and DCs were harvested on day 6 for phenotypic analysis. Results of a representative experiment assessing the co-expression of CD83 and HLA-DR (**A**) or CD40 (**B**) on control and drug-treated DCs are shown. Similar data were obtained in three independent experiments using PBMC from three different donors. Control, non-treated DCs.

FACScan analysis of the percentage of positive cells confirmed these results. For instance, Figure 1A demonstrates that paclitaxel (5 nM) increased the expression of HLA-DR on CD83+ DCs up to 155%, while bleomycin (1 nM) had no effect. Figure 1B represents the results of CD40 expression on control and drug-treated DCs and shows that methotrexate (5 nM) doubled the percentage of CD83+ DCs expressing CD40, while the effect of bleomycin (1 nM) was neglected. These data were reproduced in three independent studies.

Thus, these results demonstrate that selected chemotherapeutic drugs, including paclitaxel, methotrexate, vincristine, and doxorubicin, in low noncytotoxic concentrations may directly up-regulate phenotypic maturation of human DCs *in vitro*. This raised the question whether these chemotherapeutic agents in low concentrations might directly affect antigen-presenting function of DCs, which is known to be coupled with DC maturation.

### Chemomodulation of antigen-presenting function of DCs by chemotherapeutic agents in low noncytotoxic concentrations

**Figure 2:**
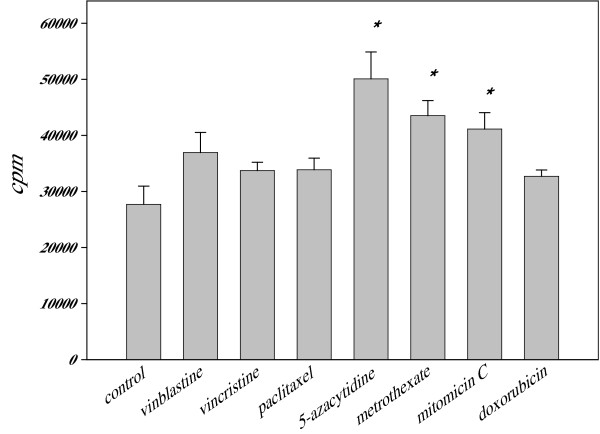
**Up-regulation of antigen-presenting function of human DCs treated with chemotherapeutic agents in low noncytotoxic concentrations**. Human monocyte-derived DCs were treated with low nontoxic concentrations of selected drugs for 48 h. Cells were collected on day 6 and co-cultured with allogeneic nylon-wool purified T lymphocytes for 96 h. Cell cultures were pulsed with ^3^H-thymidine for 4 h prior to harvesting and counting in a liquid scintillation counter. The drugs were used in the following concentrations: vinblastine and vincristine, 1 nM; paclitaxel, azadeoxycytidine, and methotrexate, 5 nM; doxorubicin, 10 nM; mitomycin C, 50 nM. The mean ± SEM. *, p < 0.05 (ANOVA, N = 4). Control, non-treated DCs.

The overall ability of DCs to present antigens is commonly tested by the allogeneic MLR assay [34]. The results of evaluation of the ability of control and drug-treated DCs to induce allogeneic T cell responses are shown in Figure 2. As demonstrated, introduction of low noncytotoxic concentrations of chemotherapeutics to DC cultures did not decrease the ability of DCs to induce proliferation of allogeneic T cells. Rather, we revealed that several agents stimulated antigen-presenting function of DCs in the MLR assay: DCs treated with 5-aza-2-deoxycytidine (10 nM), methotrexate (5 nM) and mitomycin C (50 nM) showed increased potential to stimulate T cell proliferation in comparison with untreated control DCs. For instance, in the optimal DC:T cell ratio 1:3, T cell proliferation reached 48,093 ± 2,010 cpm, 42,198 ± 769 cpm, and 40,428 ± 1,423 cpm when DCs were pre-treated with 5-azacytidine, methotrexate, and mitomycin C, respectively (p < 0.05 versus 32,362 ± 1,124 cpm for control DCs, ANOVA, N = 4). Thus, these results suggest that certain chemotherapeutic drugs in low nontoxic concentration were able to directly up-regulate antigen-presenting function of human DCs *in vitro*.

## Discussion

Antineoplastic chemotherapy agents act on highly proliferating tumor cells; however, proliferation of immune cells might be also affected by a variety of cytotoxic drugs. The suppression of the immune response by conventional high-dose chemotherapy may support tumor escape allowing the proliferation of chemoresistant variants of tumor cells. Decreasing the dose of chemotherapeutics has been suggested as an alternative approach, which might limit many side effects of conventional cytotoxic chemotherapy [35,36]. In addition, low-dose chemotherapy might support the development of immune responses against the tumor [37,38], although direct immune modulating activities of chemotherapeutic agents was not explored yet. Understanding the effect of low-dose nontoxic chemotherapy on the immune system is fundamental for improving the efficacy of immunotherapy in combinatorial anticancer modalities.

In the present study, we showed for the first time that the treatment of human DCs with different chemotherapeutic agents in very low concentrations did not induce apoptosis of DCs, but stimulated DC maturation and increased the ability of DCs to induce T cell proliferation. Our results are in agreement with the *in-vivo *data reported by Liu et al. [38] and might explain their observation that a single administration of low-dose cyclophosphamide (50 mg/kg) in tumor-bearing mice prior to immunization with DCs increased the frequency of IFN-γ secreting antitumor CTLs. In the present study, we revealed that treatment of DCs with mitomycin C, which also belongs to the family of alkylating agents as cyclophosphamide does, increased the ability of DCs to stimulate T cell proliferation. Interestingly, Jiga et al. observed that mitomycin C, when used in concentrations that are significantly higher (up to 6.0 μM) than those used in our studies, induced the generation of tolerogenic DCs, which expressed low levels of CD80 and CD86 and displayed low activity in the MLR assay [16]. Our data also differ from the results of Chao et al., who reported that doxorubicin and vinblastine significantly reduced the antigen-presenting function of human DCs assessed in the MLR assay [12]. However, the concentrations of drugs used in that study were at least 25 times higher for doxorubicin and 20,000 times higher for vinblastine then the concentrations we used in our experiments. Therefore, DCs might demonstrate diverse immunobiological responses to chemotherapy that depend on the concentration of a chemotherapeutic agent. Our data support this conclusion and demonstrate that cytotoxic agents might display unusual properties when used in ultra-low noncytotoxic concentrations: they may stimulate functional activation of human DCs *in vitro*.

The concentrations of chemotherapy agents used in our studies are lower than the therapeutic concentrations achieved in plasma in patients during chemotherapy, although the significance of this comparison is quite limited due to complex pharmacodynamics of many drugs *in vivo*. For instance, in patients receiving three consecutive 3-weekly courses of conventional paclitaxel at dose levels of 135, 175, and 225 mg/m^2^, the plasma levels of the drug reached 10.2 ± 1.34 to 15.5 ± 1.38 and 31.8 ± 5.40 μM [39]. However, administration of low-dose metronomic vinblastine (1 mg/m^2 ^IV 3×/wk) in cancer patients resulted in peak plasma concentrations of vinblastine reaching 30 μg/l, i.e. ~37 nM [40]. To the best of our knowledge, this constitutes the first report of low-dose vinblastine pharmacokinetics in any human population. These nanomolar concentrations were slightly higher than the concentrations used in our studies, but were in a close range. Interestingly, in the abovementioned group of patients treated with low-dose metronomic vinblastine, the plasma concentrations measured were above the preclinically validated target concentration of 1 pM, as was estimated based on the effect of vinblastine on angiogenesis in vivo in the chick embryo chorioallantoic membrane (CAM) model [41].

The dose-dependent immunomodulating activities of chemotherapeutic agents were also reported for other immune cell populations. For instance, cyclophosphamide might not only decrease the number and proliferation of regulatory T cells (Treg), but also down regulate their function [42]. Recently, Banissi et al. reported that administration of low dose of temozolomide in glioblastoma-bearing rats significantly decreased the number of Treg, whereas a high-dose regimen did not modify the number of these cells [43]. Furthermore, Tanaka et al. have used an experimental model to study a combination of intratumoral injection of DCs with chemotherapeutic agents where MC38-bearing mice were treated i.p. with 5-fluoracil and cisplatin [44]. The authors observed that the high doses of drugs (100 mg/kg 5-FU + 1.0 mg/kg CIS), which were needed for inhibiting tumor growth, were also lethal for all animals. While the lower doses of drugs (10 mg/kg 5-FU+ 0.1 mg/kg CIS) only delayed the tumor growth during the first week, the combination of low-dose chemotherapy with intratumoral inoculation of DCs completely abrogated tumor growth in mice. Similarly, we have recently reported that a single administration of low-dose paclitaxel prior to intratumoral DC vaccine in 3LL-bearing mice caused a significantly stronger inhibition of tumor growth than either therapy alone [28]. Low nontoxic concentrations of paclitaxel were not only able to up-regulate function of murine DCs, but protected DCs from tumor-induced inhibition [28]. Thus, although moderately low doses of certain chemotherapeutic agents could indirectly support antitumor immunity by blocking Treg- or myeloid-derived suppressor cells (MDSC)-mediated immune tolerance or activating DCs by "danger" signals released from dying tumor cells [23,36,45], it seems that the use of lower doses of cytotoxic drugs, i.e. low-dose noncytotoxic chemomodulation, might represent a new approach for altering immunogenicity of the tumor microenvironment and improving the antitumor potential of both resident DCs and exogenous DCs administered as a vaccine.

The effects of methotrexate, paclitaxel, vincristine, and vinblastine on maturation and activation of human DCs additionally supports the feasibility of adjuvant chemomodulation or chemo-immunotherapy, since these drugs were able to increase the level of expression of CD83, CD80, and especially CD40 on DCs. CD40 is a phospholipoprotein belonging to the superfamily of type I TNF-receptors that expressed on both normal host cells (mainly DCs, B lymphocytes, macrophages and mast cells) and some tumor cells [46]. Expression of CD40 on DCs is essential for their interaction with T lymphocytes and development of efficient Th1 responses [47]. Because expression of CD40 on DCs, as well as CD40-mediated DC function are suppressed during tumor progression [48], its up-regulation by nontoxic chemotherapy should support the development of antitumor immunity in tumor-bearing hosts. In addition, CD40 ligation protects human and murine DCs from tumor-induced apoptosis by inducing expression of anti-apoptotic proteins from the Bcl-2 family [32,49,50].

Increased expression of CD40 molecules on DCs treated with methotrexate and mitomycin C is in agreement with their increased ability to stimulate T cell proliferation in the MLR assay (Figure 2). However, this correlation was not seen for other tested drugs, suggesting the importance of other mechanisms involved in up-regulation of antigen-presenting function of DCs by chemomodulation. In fact, in the murine models, we have recently revealed that the ability of DCs treated with paclitaxel, methotrexate, doxorubicin, and vinblastine to increase antigen presentation to antigen-specific T cells was abolished in DCs generated from IL-12 knockout mice, indicating that up-regulation of antigen presentation by DCs is IL-12-dependent and mediated by the autocrine or paracrine mechanisms. At the same time, IL-12 knockout and wild type DCs demonstrated similar capacity to up-regulate antigen presentation after their pretreatment with low concentrations of mitomycin C and vincristine, suggesting that these agents do not utilize IL-12-mediated pathways in DCs for stimulating antigen presentation [22].

In summary, our results show for the first time that several FDA-approved antineoplastic chemotherapeutic agents in low noncytotoxic concentrations do not reduce longevity and activity of normal human DCs; conversely, this treatment, i.e. chemomodulation, promotes maturation of DCs and their antigen-presenting activity. These data thus provide evidence that chemomodulation might be used for the generation of effective DC vaccines *ex vivo *and for improving function of resident DCs *in vivo *in the diseases associated with inhibited functionality of conventional DCs, e.g., cancer. These results also support further studies to evaluate the feasibility and clinical applicability of using chemomodulation of human DCs *in vivo*.

## Conclusion

Our data demonstrate for the first time that in low noncytotoxic concentrations chemotherapeutic agents do not induce apoptosis of human DCs, but directly enhance DC maturation and function. This suggests that modulation of human DCs by noncytotoxic concentrations of antineoplastic drugs, i.e. chemomodulation, represents a novel approach for up-regulation of functional activity of resident DCs in the tumor microenvironment or improving the efficacy of DCs prepared *ex vivo *for subsequent vaccinations.

## Competing interests

The authors declare that they have no competing interests.

## Authors' contributions

RK carried out the functional studies and flow cytometry, performed the statistical analysis and drafted the manuscript. GVS carried out drug titration experiments and supervised all flow cytometry analyses. ILT participated in cell viability studies and performed many pilot experiments. MRS conceived of the study, participated in its design and coordination and edited the manuscript. All authors read and approved the final manuscript.
